# A Case Presenting with Neuromyelitis Optica Spectrum Disorder and Infectious Polyradiculitis Following BNT162b2 Vaccination and COVID-19

**DOI:** 10.3390/vaccines10071028

**Published:** 2022-06-27

**Authors:** Youngho Kim, Donghyun Heo, Moonjeong Choi, Jong-Mok Lee

**Affiliations:** 1 Department of Neurology, Kyungpook National University Hospital, 130, Dongdeok-ro, Jung-gu, Daegu 41944, Korea; youngho1022@gmail.com; 2 School of Medicine, Kyungpook National University, 680, Gukchaebosang-ro, Jung-gu, Daegu 41944, Korea; dhheo0525@naver.com (D.H.); jennyjade@naver.com (M.C.); 3 Department of Neurology, School of Medicine, Kyungpook National University, 680, Gukchaebosang-ro, Jung-gu, Daegu 41944, Korea

**Keywords:** SARS-CoV-2, polyradiculopathy, virus disease, anti-aquaporin-4 autoantibody, acute transverse myelitis, neuromyelitis optica spectrum disorder

## Abstract

A 37-year-old woman presented with paraparesis and paresthesia in both legs 19 and 3 days after BNT162b2 vaccination and severe acute respiratory syndrome coronavirus 2 (SARS-CoV-2) infection, respectively. Cerebrospinal fluid (CSF) analysis, nerve conduction study, electromyography, magnetic resonance imaging, and autoantibody tests were performed. Neurological examination showed hyperesthesia below the T7 level and markedly impaired bilateral leg weakness with absent deep tendon reflexes on the knees and ankles. CSF examination revealed polymorphonuclear dominant pleocytosis and elevated total protein levels. Enhancement of the pia mater in the lumbar spinal cord and positive sharp waves in the lumbar paraspinal muscles indicated infectious polyradiculitis. In contrast, a high signal intensity of intramedullary spinal cord on a T2-weighted image from C1 to conus medullaris and positive anti-aquaporin-4 antibody confirmed neuromyelitis optica spectrum disorder (NMOSD). The patient received intravenous methylprednisolone, antiviral agents, and antibiotics, followed by a tapering dose of oral prednisolone and azathioprine. Two months after treatment, she was ambulatory without assistance. The dual pathomechanism of NMOSD triggered by coronavirus disease 2019 (COVID-19) vaccination and polyradiculitis caused by SARS-CoV-2 infection may have caused atypical clinical findings in our patient. Therefore, physicians should remain alert and avoid overlooking the possibilities of diverse mechanisms associated with neurological manifestations after SARS-CoV-2 infection and COVID-19 vaccination.

## 1. Introduction

Neuromyelitis optica (NMO), also known as neuromyelitis optica spectrum disorder (NMOSD), is a well-known central demyelinating disease. Traditionally, NMO has been diagnosed based on the simultaneous involvement of bilateral optic neuritis and transverse myelitis [1]. However, relapsing cases began to be introduced, and serum antibodies against aquaporin-4 (AQP4) water channels were discovered in most patients with NMO, which widened its clinical spectrum [1]. Currently, NMOSD is diagnosed based on the presence of anti-AQP4-IgG with at least one core clinical characteristic: optic neuritis, acute myelitis, area postrema syndrome, or acute brainstem syndrome [1]. Although the precise pathogenesis of NMOSD is not yet understood, NMOSD has been reported following many infectious diseases, including those caused by varicella-zoster virus, Epstein–Barr virus, herpes simplex virus, cytomegalovirus, and *Mycobacterium pneumoniae* as well as tuberculosis [2]. NMOSD has also been reported to be associated with coronavirus disease 2019 (COVID-19) [2].

Since the outbreak of COVID-19, the disease or its vaccination has affected the bronchopulmonary system and can manifest in nearly all compartments of the body, including the central and peripheral nervous systems [3]. Severe acute respiratory syndrome coronavirus 2 (SARS-CoV-2) is an immune trigger associated with bystander activation, epitope spreading, and molecular mimicry [2]. Subsequently, anti-AQP4-IgG antibodies and inflammatory cells infiltrating susceptible hosts can cause NMOSD [2]. Regarding the peripheral nervous system, an association between SARS-CoV-2 and polyradiculitis has been reported [3]. Most patients suffer from immune-mediated polyradiculitis without evident pleocytosis in the cerebrospinal fluid (CSF) [3,4]. To the best of our knowledge, infectious polyradiculitis caused by SARS-CoV-2 has not yet been reported. Here, we report the case of a 37-year-old woman who presented with acute transverse myelitis (ATM), NMOSD, and infectious polyradiculitis due to COVID-19 and BNT162b2 vaccination.

## 2. Case

**Figure 1:**
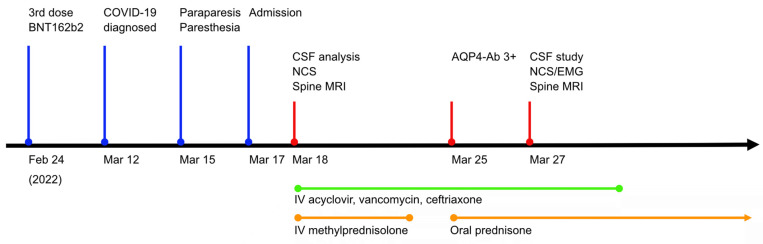
Timeline of clinical date, investigation, and medication. Clinical presentation and administration of proper medication were depicted on the appropriate date. AQP4-Ab, anti-aquaporin 4 antibody; COVID-19, coronavirus disease 2019; CSF, cerebrospinal fluid; NCS, nerve conduction study; MRI, magnetic resonance imaging.

A 37-year-old woman visited the emergency room presenting with rapidly progressive bilateral leg weakness for two days. She denied any previous obstetric and drug history or other underlying neurological diseases. The patient had no apparent family history of neurological disorders. One month previously, she had been vaccinated with a COVID-19 booster of BNT162b2 and did not experience noticeable side effects. The patient had been vaccinated with mRNA-1273 twice before. However, she was infected with COVID-19 the day after her 40-month-old son became infected and tested positive for SARS-CoV-2 polymerase chain reaction (PCR) conducted from a nasal swab five days before the patient’s admission. She did not complain of any other symptoms related to the direct SARS-CoV-2 infection, including upper airway symptoms or fever. Two days before admission, she experienced weakness and a tingling sensation on both legs with urinary incontinence and constipation (Figure 1).

On neurological examination performed at admission, bilateral leg weakness was noted. Specifically, as per the Medical Research Council (MRC) standard, hip flexion was two, and ankle dorsiflexion was four. Weakness in other movements of the bilateral leg was three. Hyperesthesia was identified bilaterally below the T7 level. The deep tendon reflex (DTR) was mute on the knees and ankles, but normal on the brachioradialis and biceps. The extended disability status scale score was 8.5.

**Figure 2:**
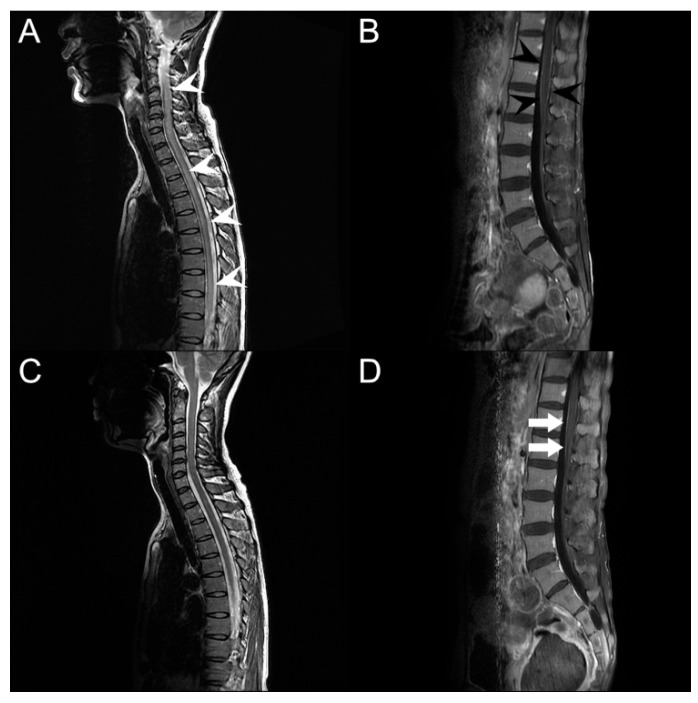
Spine magnetic resonance imaging. (**A**) T2-weighted sagittal imaging on admission shows abnormal intramedullary hyperintensity lesions from C1/2 to conus medullaris (white arrowheads). (**B**) T1-weighted sagittal gadolinium-enhanced imaging shows enhancement of pia mater on the conus medullaris (black arrowheads). (**C**) Intramedullary hyperintensity is improved after treatment. (**D**) Enhancement of pia mater remains even after treatment (white arrows).

Routine blood tests performed on the day of admission showed an elevated C-reactive protein (CRP) level (7.09; normal range < 0.5) and erythrocyte sedimentation rate (74 mm/h; normal range 0–20 mm/h). Venereal disease research laboratory (VDRL) test, human immunodeficiency virus (HIV) antigen, and antibody tests were negative. Other laboratory results, including vitamins B1, B6, B12, methylmalonic acid, thyroid-stimulating hormone, T3, hemoglobin A1c, Jo-1, SS-A/Ro, SS-B/La, double-stranded DNA, paraneoplastic antibodies, and anti-ganglioside antibodies, were within the normal range. The opening pressure of the lumbar puncture for CSF analysis was 11 cmH_2_O with a clear color. However, CSF testing showed polymorphonuclear dominant (73.6%) pleocytosis (total white blood cell count 602/µL; normal range 0–5/µL) with elevated levels of protein (188.4 mg/dL; normal range 15–45 mg/dL). The CSF VDRL test results were also negative. The IgG index increased slightly to 0.98, with a negative oligoclonal band. Other specific infection markers, including CMV, *Mycobacterium tuberculosis*, *Mycoplasma pneumoniae*, varicella-zoster virus, herpes simplex virus type I and II, *Streptococcus pneumoniae*, *Neisseria meningitidis*, *Hemophilus influenzae* type 1, *Listeria monocytogenes*, Group B streptococcus, and *Cryptococcus*, were negative in the CSF. Serum and urine immunofixation did not reveal any abnormal bands. The PCR test for SARS-CoV-2 in the CSF was negative. No pathogenic bacteria were cultured from the blood, urine, sputum, or CSF. The results of the nerve conduction study (NCS) were normal. Magnetic resonance imaging (MRI) revealed a high signal intensity in the intramedullary spinal cord from C1 to the conus medullaris on a T2-weighted image (Figure 2A). The signal was also highlighted in the T11 to L2 level pia mater on an enhanced T1-weighted image (Figure 2B). However, diffusion-weighted imaging (DWI) did not reveal any abnormal signal changes. These findings led us to suspect immune-triggered transverse myelitis and infectious polyradiculitis. We started administering intravenous steroid (methylprednisolone 1 g daily) for five days and an antiviral agent (acyclovir 30 mg/kg daily) and antibiotics (ceftriaxone 4 g and vancomycin 2 g daily) for 14 days (Figure 1) to the patient.

Five days after drug administration with no adverse events observed, NMOSD was diagnosed based on the positivity of the anti-AQP4 antibody. Intravenous methylprednisolone was replaced by tapering oral prednisolone and azathioprine dosage for maintenance therapy.

Seven days after starting medication, the bilateral leg weakness began to improve to grade 4, and the knee jerk was 1+ with a positive Babinski sign on the left. Sensory disturbance improved bilaterally below the T7 level. In the follow-up evaluation, NCS identified prolonged gastrocnemius-soleus H-reflexes, and needle electromyography (EMG) showed positive sharp waves on the thoracic and lumbosacral paraspinal muscles, which indicated polyradiculopathy. Follow-up blood and CSF tests revealed remarkable improvement. The serum CRP level returned to the normal range without leukocytosis, and the WBC count and protein level in the CSF were normal at 4/µL and 33.1 mg/dL, respectively. The follow-up infection markers, including PCR for SARS-CoV-2 in the CSF, were still negative. There was no noticeable growth in the blood, CSF, urine, or sputum cultures. Moreover, MRI revealed a normal T2-weighted signal intensity in the spinal cord (Figure 2C). Nevertheless, the enhancement of the pia mater located at the conus medullaris remained (Figure 2D). Two months after admission, the patient’s overall neurological manifestations recovered. Motor weakness improved to 5 in the MRC grading system, except for the bilateral proximal leg, which was graded as 4+, which allowed her to ambulate without assistance. The sensory level decreased from T7 to L1, and the DTR returned to normal. However, the patient still complained of mild voiding difficulties and constipation.

## 3. Discussion

Initially, we suspected Guillain–Barré syndrome (GBS) or spinal cord infarction based on the following findings: acute progressive leg weakness, diminished DTR, sensory disturbances, and multiple triggering factors, including vaccination and acute respiratory infection. The later results of DWI and anti-ganglioside antibodies were negative, suggesting a low possibility of spinal cord infarction or GBS. However, we raised the possibility of infectious polyradiculitis following SARS-CoV-2 infection on identifying marked polymorphonuclear dominant pleocytosis in the CSF, the extent of which was higher than that generally accepted as a demyelinating disease. In addition, the diagnosis of polyradiculitis was supported by the decreased DTR, the findings of NCS and EMG rendering radiculopathy, and the enhancement of the spinal cord pia mater later. The presence of the Babinski sign, serum anti-AQP4 antibody positivity, and abnormality on spine MRI confirmed NMOSD. Therefore, both NMOSD and infectious polyradiculitis appeared to have acted simultaneously in our patient.

Both trigger events, SARS-CoV-2 infection and COVID-19 vaccination, were not remote from the onset of weakness or paresthesia. It is difficult to define which event caused these neurological symptoms. In previously reported cases of newly diagnosed NMOSD following COVID-19 vaccination, the duration from vaccination to clinical onset ranged from five days to four weeks [2]. Considering that the duration from vaccination to clinical onset was 19 days in our patient, NMOSD was presumed to be caused by the BNT162b2 injection rather than SARS-CoV-2 infection. Although SARS-CoV-2 PCR results in the CSF were negative, polyradiculitis was speculated to be associated with SARS-CoV-2 infection. Pleocytosis, an elevated level of protein in CSF, and the short interval between polyradiculitis and SARS-CoV-2 infection supported our speculation.

Both infectious polyradiculitis and NMOSD can cause paraparesis and paresthesia, but it is difficult to define which mechanism is predominantly responsible for the clinical presentation. To the best of our knowledge, viral polyradiculitis appears to play a role in the development of the symptoms. First, abnormal spontaneous activity was not observed in the cervical paraspinal muscles during needle EMG. Second, the muscle strength of the upper extremities remained normal despite the involvement of the cervical cord. Third, the gadolinium enhancement of the pia mater was limited to the lower thoracic region and conus medullaris.

The association of post-vaccination NMOSD has been reported in cases of influenza, tetanus/diphtheria, pneumococcal, hepatitis, human papillomavirus, and Japanese encephalitis vaccination as well as for mRNA-based vaccines for COVID-19 [5,6,7,8]. NMOSD following vaccination is a rare condition, and only a few cases have been reported to date [5,6,7]. Although the relationship between NMOSD and vaccination is not fully understood, it is possibly attributed to a certain level of pre-existing systemic immunity, which overactivates the immune system and exacerbates the production of anti-AQP4-IgG [5,6,7]. Furthermore, patients with NMOSD and non-neurological symptoms had significantly higher serum anti-AQP4-IgG titers than those without neurological symptoms [9]. These findings can be explained by the upregulation of autoimmunity owing to an inflammatory response to infectious agents. Vaccines have also been developed to boost the host immune response by imitating certain steps in the process of natural infection [9]. Therefore, NMOSD following vaccination may be explained by similar mechanisms. Clinically, the location of lesions in NMOSD following COVID-19 vaccination includes the cervical spinal cord and ependymal surface of the lateral or third ventricles [5,6,7]. All patients showed improvement in neurological symptoms [5,6,7].

Concerning the relationship between NMOSD and COVID-19, patients with NMOSD following COVID-19 are rare, and some studies have been reported in this regard [6,10]. Most of these patients experience visual loss [10]. In addition to neurological symptoms, fever and respiratory symptoms are also common [10]. A possible mechanism of NMOSD following COVID-19 includes SARS-CoV-2 invasion into the central nervous system (CNS) via the trans-synaptic route of the olfactory bulb and its ability to cross the blood–brain barrier [11,12]. The mimicry property between SARS-CoV-2 and the dorsal motor nucleus, nucleus ambiguous, and nodose ganglion sharing 22 proteins could also be a reason [10]. Therefore, increasing Th1, Th2, and natural killer cell numbers and cytokine levels can lead to an autoimmune event [10]. The prognosis of NMOSD following COVID-19 is good in patients under 55 years of age [10]. In a previous study, 8 of 11 patients showed improvement, whereas 2 patients aged >55 years died [10].

Polyradiculitis is a manifestation of post-COVID-19 or COVID-19 vaccination [3,4]. Clinical presentations of post-infection/vaccination polyradiculitis include GBS, Miller–Fisher syndrome, acute motor axonal neuropathy, and acute motor-sensory axonal neuropathy [3,4]. However, its pathomechanism remains unclear. Polyradiculitis is triggered by an immune-mediated pathway or by molecular mimicry [3,4]. The duration of latency between onset and SARS-CoV-2 infection/COVID-19 vaccination ranged from 1 to 23 days, except for outliers (3 h and 39 days). The prognosis of the reported cases was favorable; however, several patients required mechanical ventilation or recovered partially [3,4].

Infectious polyradiculitis is common in patients with acquired immunodeficiency syndrome, with CMV being the most common opportunistic pathogen [13]. CSF analysis findings of CMV polyradiculitis include polymorphonuclear pleocytosis and an elevated protein level, resembling bacterial meningitis [13]. Although CSF PCR results for HIV, CMV, and SARS-CoV-2 were negative in our patient, the elevated protein level and polymorphonuclear pleocytosis in CSF were similar to those previously reported, which supports the diagnosis of viral polyradiculitis in our patient [13]. The enhancement of the pia mater on the conus medullaris on T1-weighted sagittal gadolinium-enhanced imaging also supported the diagnosis of infectious polyradiculitis (Figure 2C,D).

Negative SARS-CoV-2 in the CSF could be a limitation of our explanation for our patient’s SARS-CoV-2-associated infectious polyradiculitis. In a previous study of neurological manifestations related to COVID-19, there were diverse profiles of CSF studies, but only 2 out of 58 cases were positive for SARS-CoV-2 RNA [14]. This is likely because of lower SARS-CoV-2 RNA copies per cell in the brain tissue than in the lungs or pharynx tissues, or transient or limited SARS-CoV-2 dissemination in the CNS [14]. Therefore, we attributed polyradiculitis to SARS-CoV-2, although the CSF PCR results were negative.

The treatment of NMOSD following SARS-CoV-2 infection or COVID-19 vaccination did not differ from that of NMOSD without comorbidities [2]. The acute treatment of NMOSD following SARS-CoV-2 infection or COVID-19 vaccination comprises intravenous methylprednisolone and plasmapheresis [2,5,6,7,8]. Most reported cases improved in the acute phase [2,5,6,7,8], and mild residual symptoms were complicated [5].

At present, most people worldwide are trying to return to normal daily life post-COVID-19. However, the threat of SARS-CoV-2 remains, regardless of its severity. In a meta-analysis review, up to one-third of the patients with COVID-19 experienced at least one neurological manifestation. Therefore, we should inspect COVID-19-related personal histories and assume the possibility of a relationship between neurological diseases and COVID-19. Finally, physicians should remain alert and should not overlook the neurological manifestations of COVID-19 infection or vaccination.

## 4. Conclusions

Given the COVID-19 pandemic and the wide application of COVID-19 vaccines, there are many patients with unexpected neurological manifestations. Here, we reported an unusual case of infective polyradiculitis caused by SARS-CoV-2 infection combined with immune-associated NMOSD—a case that has not been reported before. This report highlights the autoimmune adverse events following SARS-CoV-2 infection and COVID-19 vaccination as well as presents the diverse mechanisms between neurological manifestations and COVID-19 and/or its vaccination.

## Data Availability

Not applicable.
